# The influence of stress path and flaw morphology on the failure mechanism and mechanical properties of rock masses

**DOI:** 10.1371/journal.pone.0349190

**Published:** 2026-05-14

**Authors:** Na Wu, Yu Gan, Jie Hu, Ruixiang Sun, Hongyan Zeng

**Affiliations:** 1 College of Civil Engineering and Architecture, Dalian University, Dalian, China; 2 Nanjing Yangtze River Management Office, Nanjing, China; 3 State Key Laboratory for Tunnel Engineering, China University of Mining and Technology-Beijing, Beijing, China; 4 International Scientific and Technological Cooperation Base for Geological Disaster Prevention of Zhejiang Province, College of Civil Engineering, Shaoxing University, Shaoxing, China; China Construction Fourth Engineering Division Corp. Ltd, CHINA

## Abstract

The macroscopic failure of rock masses is essentially the result of the propagation, interaction, and eventual coalescence of internal defects such as flaws under stress. Given the widespread presence of three-dimensional (3D) flaws in rock mass engineering, it is crucial to investigate their failure mechanisms and mechanical properties. This research employs a meso-damage numerical simulation method to systematically investigate the failure process and mechanical parameters of heterogeneous rock masses containing surface flaws, through-going flaws, and internal flaws under various stresses, focusing on the influence of stress path on flaw propagation behavior and the mechanical properties of the specimens. The study reveals: (1) Rock mass flaw propagation primarily manifests as the extension of wing and anti-wing flaws, and the coalescence of secondary flaws with these or with the pre-existing flaw. (2) Under uniaxial compression test, conventional triaxial and true triaxial compression test with specific intermediate principal stress directions, rock masses exhibit tensile coalescence failure, while changing the intermediate principal stress direction leads to transverse coalescence failure. The direction of the intermediate principal stress plays a decisive role in flaw propagation path. (3) Confining pressure significantly enhances the peak strength of the rock mass. For embedded and through-going flaws, the peak strength is generally higher when the intermediate principal stress direction is oriented towards the flaw plane compared to when it is parallel. Whereas the peak strength of surface flaws is not significantly affected by the intermediate principal stress direction. (4) The magnitude of the intermediate principal stress affects the mechanical properties of rock masses with different flaw types to varying degrees. The results of this study can provide valuable references for theoretical research and physical experiments on the propagation mechanisms of 3D flaws in rock masses.

## 1. Introduction

Rock masses are not intact continuous media; they contain various widespread defects such as joints and flaws. The macroscopic mechanical behavior and failure essence of rock masses are complex processes involving the propagation, interaction, and eventual aggregation and coalescence of these defects under external load [1–3]. In real geological and engineering environments, flaws mostly exist in three-dimensional forms, which can be categorized based on their spatial occurrence into through-going flaws, surface flaws, and internal flaws. Due to differences in their geometric morphology and spatial position, the propagation evolution laws of these three types of flaws in a triaxial stress field and their impact on the overall stability of the rock mass are significantly different [4–6]. Furthermore, studies have shown that complex stress paths also have a significant impact on the mechanical properties and failure modes of rock masses [7–8]. Therefore, a systematic comparative study of the propagation and coalescence mechanisms of different types of 3D flaws is of great theoretical and practical significance for accurately evaluating the long-term stability and safety of rock mass engineering.

In recent years, numerous research efforts have been conducted domestically and internationally focusing on the problem of 3D flaw propagation based on physical test. For surface and through-going flaws, which are relatively easy to fabricate and observe, physical experimental research has been relatively in-depth. Uniaxial and biaxial compression tests using prefabricated double fractured sandstone were conducted by Lei et al. [9–10] conducted. Using DIC technology to monitor the changes in strain field on the surface of the sample and the development process of secondary damage during the experiment. Zhou et al. [11] through true triaxial loading tests, revealed the controlling effect of pre-existing flaw morphology and confining pressure application direction on the final failure mode of sandstone specimens containing two through-going flaws, pointing out that high confining pressure enhances inter-granular interaction, thereby inhibiting flaw development and increasing specimen strength. The physical properties and fracture coalescence behavior of the red sandstone with non-parallel double fractures was studied by Yang et al. [12] under uniaxial compression. Research has shown that the strength of fractured red sandstone is weaker than that of intact samples, which is related to the inclination angle of the fractures. However, for embedded 3D flaws, physical experiment construction and direct observation are extremely difficult due to the opacity of rock materials and the challenges in sample preparation. To address this, some scholars have used transparent or homogeneous quasi-brittle materials such as glass or resin as substitutes for observation. The primary methods for fabricating internal cracks include the cut-and-paste method [13], the pre-embedding casting method [14], and the 3D printing method [15]. A significant body of research has been conducted using the pre-embedding method to investigate internal crack propagation in transparent rock-like materials, with substantial findings reported by Handin et al. [16–18]. However, problems such as the low brittleness of the polymerized resin and the non-authentic nature of metal sheets as real cracks persist. 3D printing, a promising new technology, has been employed to create transparent rock-like samples with internal cracks for propagation studies by Wang et al. [19–21]. Its advantages include digital modeling and easy control of crack geometry. However, limitations persist, such as the low brittleness of resin printing materials and the fact that the printed cracks are not genuine fractures formed by mechanical forces.

However, flaw propagation laws in such homogeneous materials are fundamentally different from those in highly heterogeneous natural rock masses. To directly observe internal flaws in rock masses, advanced techniques like Computed Tomography (CT) have been introduced. An advanced in-situ micro-computed tomography system was employed by Shao et al. [22] to quantitatively analyze and visualize the crack behavior in rock-like specimens containing pre-existing flaws. Three-dimensional models reconstructed from 2D CT images obtained at different deformation stages allowed clear identification of crack patterns in the 3D-printed specimens.

Owing to progress in computer technology, numerical simulation has emerged as a powerful pathway to explore the failure mechanisms and mechanical properties of three-dimensional fractured rock masses. Zhang et al. [23] employed the discrete element method (DEM) to investigate the mechanical properties and failure characteristics of marble containing fissures under true triaxial stress conditions and proposed a new stress path. The study revealed that the peak strength and elastic modulus of the marble specimens with fissures increased with the loading stress ratio and the initial confining pressure. The computational results demonstrated that the initial confining pressure had a significant impact on the mechanical behavior of the specimens. Mondala et al. [24] utilized an unstructured numerical simulation method to conduct uniaxial compression tests on rock specimens containing surface fissures. When the loading direction was more aligned with the pre-existing fissures, the peak stress increased further, and there was a greater likelihood that damage and failure of the specimen would occur in areas with larger pre-existing fissures.

In summary, although existing research has yielded fruitful results, studies that utilize numerical calculation methods, particularly meso-damage models capable of reflecting rock mass heterogeneity, to systematically simulate and comparatively analyze the propagation processes of embedded, surface, and through-going-these three typical types of 3D flaws-under true triaxial stress paths are still insufficient. How different flaw morphologies affect the final failure mode and strength characteristics of rock masses under complex stress states (still requires in-depth discussion). Based on this, this paper aims to use meso-damage numerical analysis to systematically simulate the fracture processes of three types of 3D flaws under various triaxial stress paths, and to compare in detail the coupled effects of flaw morphology and stress path on the failure mode and mechanical parameters of rock masses, hoping to provide numerical basis and reference for related physical experiments and theoretical models.

## 2. Test samples and schemes

To comparatively analyze the failure mechanism and mechanical properties of rock masses with different types of flaws under various stress, a series of numerical models were constructed. The model size is 80 mm × 40 mm × 40 mm. The flaw dip angle is fixed at 45°. The flaw types are mainly internal flaws, surface flaws, and through-going flaws. All numerical simulations were performed using RFPA³D (Rock Failure Process Analysis), a finite element-based meso-damage software. Material heterogeneity is accounted for by assigning mechanical properties (elastic modulus, strength, etc.) to each element according to a Weibull distribution. The homogeneity index *m* controls the degree of material heterogeneity; a higher *m* indicates greater uniformity. In this study, a homogeneity index of *m* = 3 was used to represent natural rock heterogeneity. The mechanical parameters for each element are randomly assigned following the Weibull distribution, with the mean values derived from the macroscopic properties listed in Table 1.

**Table 1:** Mechanical parameters of rock in the numerical simulation.

Material	Homogeneity	Elastic Modulus	Uniaxial compressive strength	Poisson’s Ratio	Friction Angle	Cohesion
Rock	3	4.73 GPa	67.76 MPa	0.189	38.9°	17.44 MPa

**Fig 1:**
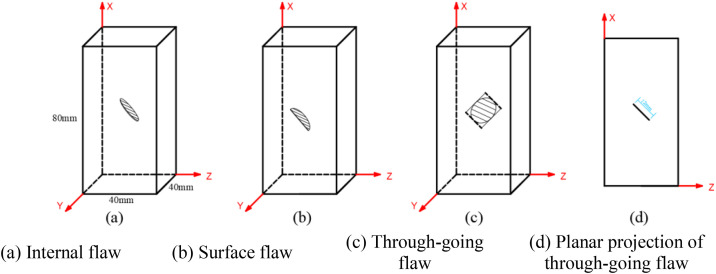
Rock specimens containing different flaws.

The flaw shape is elliptical or semi-elliptical, with the long axis of the flaw parallel to the Y-axis, as shown in Fig 1. Among them, the rock specimen with an internal flaw is shown in Fig 1(a), the flaw is semi-elliptical, with the ellipse major axis being 12 mm, the minor axis 8 mm, and the flaw width 0.5 mm. The rock specimen containing a surface flaw is shown in Fig 1(b), the surface flaw is semi-embedded elliptical, with a surface length of 12 mm, a depth of 4 mm, and a width of 0.5 mm. The rock specimen with a through-going flaw is shown in Figs 1(c) and 1(d), the flaw is elliptical, with the ellipse major axis being 40 mm, the minor axis 12 mm, and the flaw width 0.5 mm.

The distances from the flaw tips to the nearest boundaries were approximately 34 mm (top/bottom) and 14 mm (lateral), which are sufficiently large compared to the flaw size (major axis 12 mm) to avoid significant boundary interference. In addition, the flaw width of 0.5 mm is selected based on common practice in physical experiments on fractured rock masses, where prefabricated flaws are typically cut to a thickness of 0.3–1.0 mm using a high-pressure water jet or a thin saw blade [25–26]. This width is sufficiently small to approximate a closed flaw while remaining feasible for numerical meshing.

**Fig 2:**
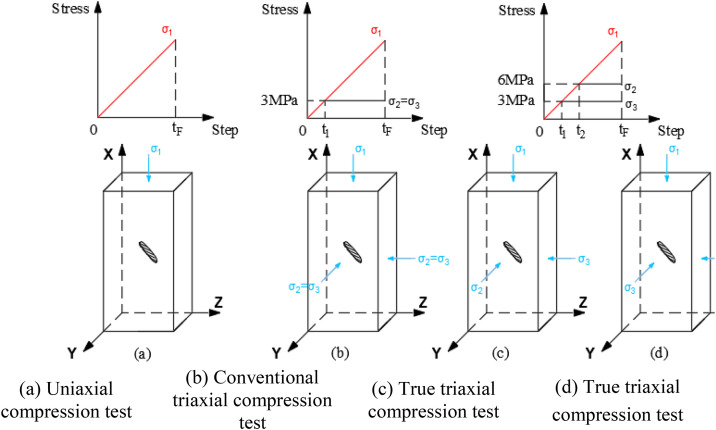
Test scheme.

To thoroughly study the influence of stress on flaw propagation process, deformation characteristics, and mechanical parameters of rock masses, numerical simulations of uniaxial compression tests (UT), conventional triaxial compression tests (CTT), and true triaxial tests (TTT) are conducted. Taking the internal flaw as an example, the specific test scheme is shown in Fig 2 and Table 2. The test schemes for surface flaws and through-going flaws are consistent with those for internal flaws.

**Table 2:** Numerical simulation test scheme.

ID	Test Type	Y-load/MPa	Z-load/MPa	X-load/mm/step
UT	Uniaxial compression test	0	0	0.01
CTT-1	Conventional triaxial compression test	3	3	
CTT-2		6	6	
CTT-3		9	9	
TTT-C1-1	True triaxial compression test	6	3	
TTT-C1-2		9	3	
TTT-C1-3		12	3	
TTT-C1-4		15	3	
TTT-C2-1		3	6	
TTT-C2-2		3	9	
TTT-C2-3		3	12	
TTT-C2-4		3	15	

For the uniaxial compression test scheme, the model bottom is fixed, and displacement load is applied to the model top at a rate of 0.01 mm/step, with the remaining surfaces being free boundaries (as shown in Fig 2(a)). For the conventional triaxial compression and true triaxial compression tests, the model bottom is fixed, confining pressure loads are applied to the other surfaces. After the confining pressure stabilizes, the top load is changed to displacement control (0.01 mm/step) until specimen failure. The true triaxial compression test scheme includes two cases: (1) The direction of the intermediate principal stress is oriented toward the flaw plane, with the loading method labeled TTT-C1 (Fig 2(c)). (2) The direction of the intermediate principal stress is oriented towards the flaw plane, with the loading method labeled TTT-C2 (Fig 2(d)).

## 3. Analysis of results for rock specimens with internal flaws

### 3.1. Influence of stress path on the failure mechanism of rock masses with internal flaws

**Fig 3:**
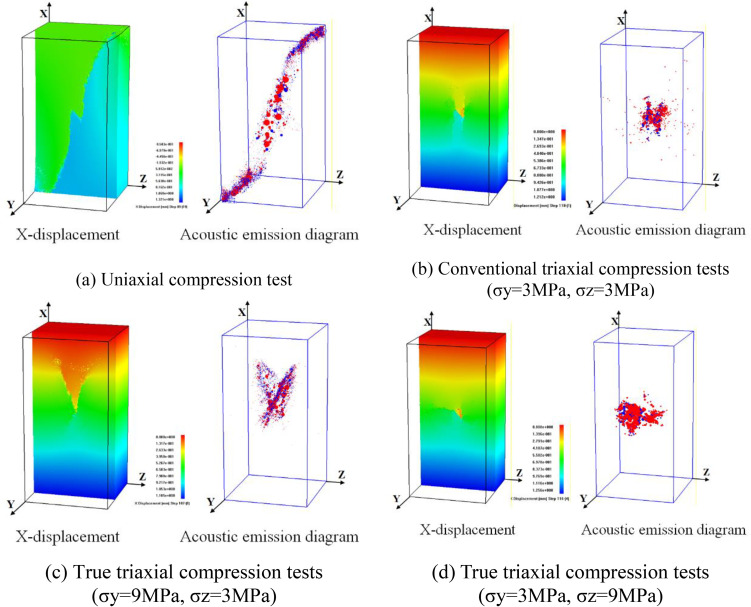
Instability failure diagrams of rock mass with internal flaws under different stress conditions.

The failure process and failure modes of rock specimens with internal flaws under different stress paths were obtained through numerical simulation. Fig 3 shows cross-sectional views of failure diagrams for some rock specimens under different stress conditions. In the numerical models, the internal flaw was positioned at the geometric center of the specimen to minimize boundary effects. The distances from the flaw tips to the nearest boundaries were approximately 34 mm (top/bottom) and 14 mm (lateral), which are sufficiently large compared to the flaw size (major axis 12 mm) to avoid significant boundary interference. The effect of flaw‑to‑boundary distance on crack propagation velocity and path will be systematically investigated in future work.

In this study, wing cracks are identified as tensile micro-cracks that initiate at the flaw tips and propagate approximately parallel to the direction of the maximum principal stress. Anti-wing cracks are also tensile in nature but propagate in the opposite direction, typically curving back toward the pre-existing flaw or toward the loading direction. The classification is based on the local stress state and the failure mode of each element, with tensile and shear failures distinguished in the acoustic emission event distribution diagrams (red for shear, blue for tensile).

Under uniaxial compression test, flaw propagation mainly manifests as shear coalescence failure dominated by anti-wing flaws. In the initial loading stage, anti-wing flaws initiate at the upper and lower tips of the internal flaw, as the axial load increases, the flaws propagate towards the specimen corners, eventually leading to shear failure of the specimen (Fig 3(a)). In the acoustic emission micro-fracture event distribution diagram, red represents shear failure and blue represents tensile failure, clearly showing the internal failure mode of the specimen.

In the conventional triaxial compression tests (Fig 3(b)), flaws first initiate and propagate from the flaw tips, forming wing and anti-wing tensile flaws. Tensile flaw branching occurs above the pre-existing flaw, leading to internal spalling. As the load increases, tensile flaws propagate along the direction of the maximum principal stress, and the specimen ultimately fails unstably. Unlike uniaxial compression test, the confining pressure restricts the flaw propagation path, and the specimen mainly exhibits tensile failure.

The true triaxial compression tests are divided into two cases: intermediate principal stress parallel to the flaw plane (TTT-C1-2) and oriented towards the flaw plane (TTT-C2-2). The former is characterized by tensile-shear failure. Wing flaws and anti-wing flaws initiate at the upper and lower tips of the cracks during the test; as the load increases, these cracks propagate along the direction of the maximum principal stress, leading to the instability of the specimen (Fig 3(c)).

The latter is dominated by compression-induced failure: anti-wing flaws initiate at the upper and lower tips of the cracks and propagate a certain distance along the direction of the maximum principal stress before arresting. Subsequently, transverse compressive cracks initiate at the upper tips of the cracks and coalesce with the side surfaces of the specimen, resulting in the instability of the specimen (Fig 3(d)).

Comparing the failure diagrams of specimens with internal flaws under different stress paths, it found that the propagation and coalescence process of internal flaws is very complex. The magnitude and direction of confining pressure have a significant influence on flaw propagation. Compared with uniaxial compression test, increased confining pressure inhibits the propagation and coalescence of flaws, and the coalescence path between the internal flaws. Moreover, the specimen surface is clearly affected by confining pressure. Compared with conventional triaxial compression tests, the application of the intermediate principal stress in true triaxial compression test is more conducive to promoting flaw propagation and coalescence.

### 3.2. Influence of stress path on the mechanical properties of rock masses with internal flaws

**Fig 4:**
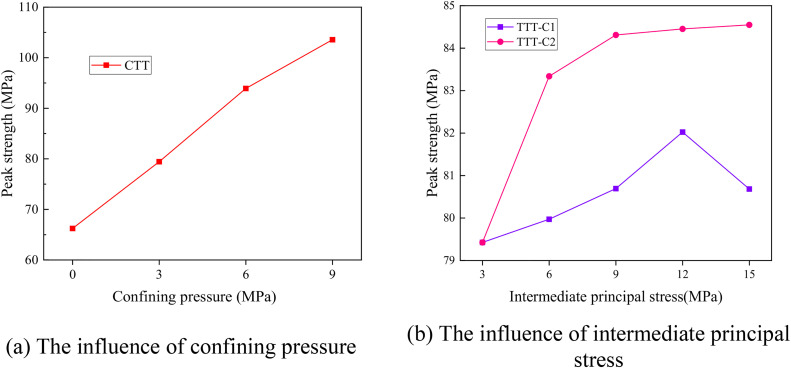
Influence of confining pressure and intermediate principal stress on the peak strength of rock mass with internal flaws under different stress conditions.

Fig 4 shows the change of peak strength of rock mass with internal flaws under different stress conditions. The complete numerical data for Fig 4 are provided in S1 File. As plotted in Fig 4(a), the peak strength of the sample increases approximately linearly with the increase of confining pressure. The result shows that confining pressure helps to enhance the ultimate strength of the specimen. The influence of intermediate principal stress on the peak strength of rock mass with internal flaws under true triaxial compression test was plotted in Fig 4(b). In the TTT-C1, when the intermediate principal stress increases from 3 MPa to 15 MPa, the peak strength of the specimen first increases and then decreases. When the intermediate principal stress increases from 3 MPa to 12 MPa, the peak strength increases by 2.82%, from 79.679 MPa to 81.929 MPa. When it further increases to 15 MPa, the peak strength decreases by 1.62% to 80.603 MPa. This indicates that when the intermediate principal stress is oriented toward the flaw plane, increasing the intermediate principal stress can enhance the peak strength of the rock mass to a certain extent, but exceeding a certain limit value will accelerate the rock mass failure process and reduce the peak strength.

After changing the direction of the intermediate principal stress (TTT-C2), when the intermediate principal stress increases from 3 MPa to 15 MPa, the peak strength of the specimen increases steadily. After the intermediate principal stress exceeds 9 MPa, the increasing trend of peak strength tends to flatten. Comparative analysis shows that when the intermediate principal stress direction is oriented towards the flaw plane, the peak strength of the specimen is significantly higher, indicating that the intermediate principal stress enhances the ultimate strength of the specimen to a certain extent.

## 4. Analysis of results for rock specimens with surface flaws

### 4.1. Influence of stress path on the failure mechanism of rock masses with surface flaws

**Fig 5:**
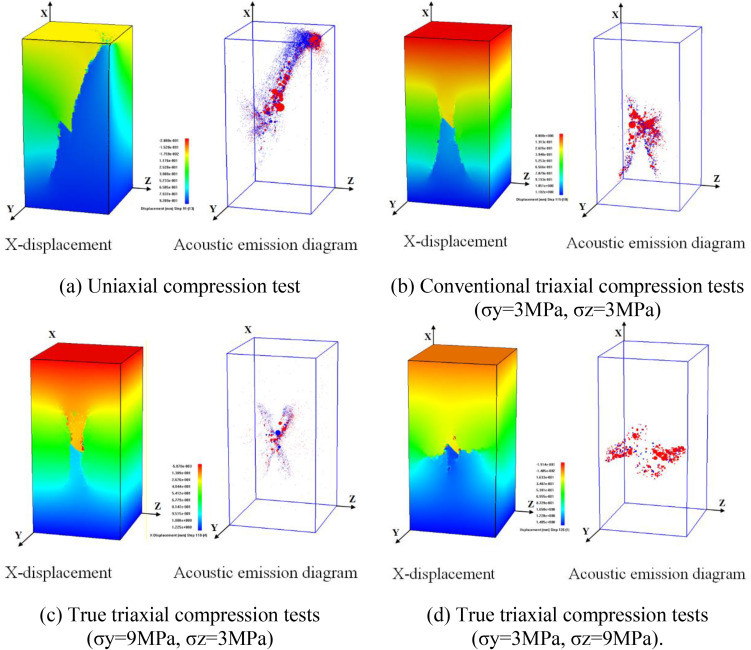
Instability failure diagrams of rock mass with surface flaws under different stress conditions.

Under uniaxial compression test, flaw propagation mainly manifests as the coalescence of anti-wing flaws leading to shear failure of the specimen, as illustrated in Fig 5(a). In the initial loading stage, anti-wing flaws initiate at the upper and lower tips of the pre-existing flaw. As the axial load increases, the flaws deviate from their original direction and propagate towards the specimen corners. The anti-wing flaws propagate approximately symmetrically, eventually coalescing with the specimen surface.

In the conventional triaxial compression model (CTT-3), flaw propagation of the rock specimen exhibits tensile-shear composite failure, as plotted in Fig 5(b). In the initial loading stage, wing flaws and anti-wing flaws initiate at the tips of the prefabricated cracks and propagate upward and downward. As the load increases, the wing and anti-wing flaws at the crack tips continue to propagate along the direction of the maximum principal stress and tend to extend toward the apexes of the model, eventually leading to the failure of the specimen.

Under TTT-C1-2, as plotted in Fig 5(c), the propagation path of the pre-existing surface flaw is partially similar to that in conventional triaxial compression test, with the main failure characteristic being tensile-shear composite failure. In the initial loading stage, wing flaws and anti-wing flaws initiate at the tips of the pre-existing flaw and propagate together. The micro-fracture event distribution diagram shows shear flaws propagating in an “X”-shaped path.

When the intermediate principal stress is oriented towards the flaw plane (TTT-C2-2), the rock specimen mainly exhibits compression-shear composite failure, as plotted in Fig 5(d). In the initial loading stage, the anti-wing flaws initiating from the pre-existing flaw tips propagate a certain distance and then stop. Subsequently, initiating tensile flaws, influenced by the intermediate principal stress, propagate transversely and eventually coalesce with the specimen’s side surface. Simultaneously, secondary flaws at the shell-like flaw also propagate, in an approximately 60° downward direction, eventually coalescing with the specimen bottom, causing model instability failure.

Comparing the results of different test schemes, confining pressure has a significant influence on surface flaw propagation, and the failure morphology of the specimens is clearly different. When the intermediate principal stress direction is consistent with the propagation direction of the secondary flaws, the intermediate principal stress significantly promotes the propagation of secondary flaws. Secondary flaws always develop rapidly and coalesce along the direction that can release stress the fastest.

### 4.2. Influence of stress path on the mechanical properties of rock masses with surface flaws

**Fig 6:**
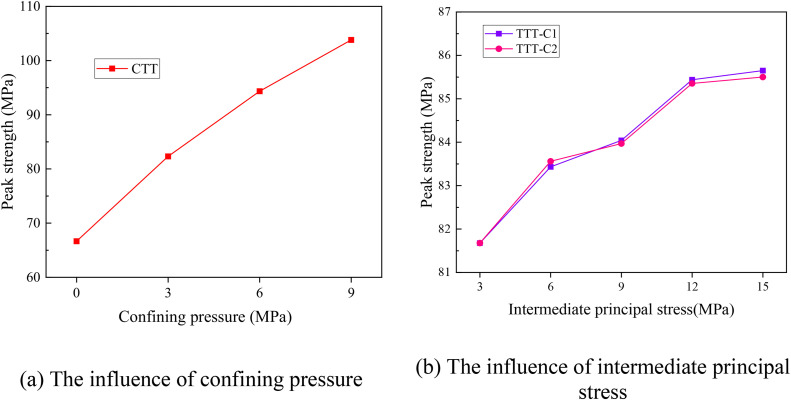
Influence of confining pressure and intermediate principal stress on the peak strength of rock mass with surface flaws under different stress conditions.

Fig 6 shows the influence of stress conditions on the peak strength of rock specimens with surface flaws. The results show that the peak strength of the specimen increases significantly by increasing confining pressure and intermediate principal stress, as plotted in Fig 6. All peak strength data for surface-flaw specimens under different confining pressures and intermediate principal stresses are available in S1 File. Furthermore, in the TTT-C, the direction of the intermediate principal stress has no significant effect on the peak strength of the specimen. In addition, in the TTT-C, the direction of the intermediate principal stress has no significant effect on the peak strength of the specimens.

## 5. Analysis of results for rock specimens with through-going flaws

### 5.1. Influence of stress path on the failure mechanism of rock masses with through-going flaws

**Fig 7:**
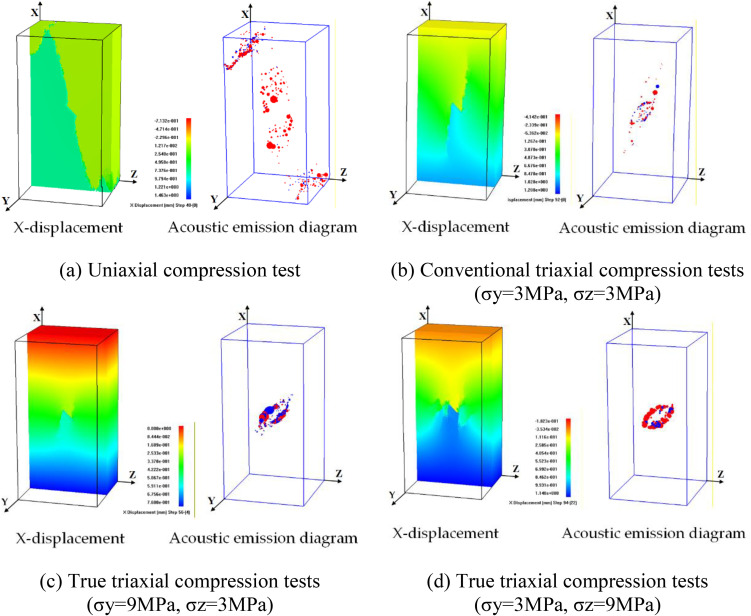
Instability failure diagrams of rock mass with through-going flaws under different stress conditions.

Under uniaxial compression, the flaw propagation of the rock specimen mainly manifests as shear failure of wing flaws, as plotted in Fig 7(a). In the initial loading stage, wing flaws initiate oriented towards the upper and lower tips of the pre-existing flaw. As the axial load increases, the wing flaws propagate along the direction of the maximum principal stress, and tilt again after a certain distance, eventually coalescing with the upper and lower surfaces. Compared with other flaw types, the internal flaw propagation spatial morphology of specimens with through-going flaws is simple, and the wing flaw propagation direction is clear. Different cross-sections show a unified flaw propagation path, forming a complete fracture surface extending upwards and downwards from the pre-existing flaw plane.

At the initial stage of conventional triaxial compression (CTT-3), multiple secondary cracks initiate near the tips of the prefabricated cracks. These secondary cracks are generally characterized as anti-wing flaws, which coalesce with the prefabricated cracks after propagation. Under the influence of confining pressure, the anti-wing flaws form a failure surface during propagation that does not coalesce with the upper and lower surfaces of the specimen, eventually leading to tensile instability and failure of the specimen, as plotted in Fig 7(b).

When the intermediate principal stress is oriented toward the flaw plane, the flaw propagation of the rock specimen exhibits compression-shear composite failure, as plotted in Fig 7(c). The central cross-section shows that the initial flaw propagation is similar to uniaxial compression test, including the propagation and arrest of wing flaws, and the propagation and coalescence of anti-wing flaws. Other cross-sections show significant differences: cross-sections (close to the side surface) show no wing flaw generation, only the central region has wing flaw initiation. Changing the cross-section direction reveals that not all anti-wing flaws from the pre-existing flaw extend up and down along the maximum principal stress direction to the end faces. Secondary flaws near the side surface deviate from their original propagation direction and coalesce with the side surface.

When the intermediate principal stress is oriented towards the flaw plane, the flaw propagation of the rock specimen is dominated by compression-shear composite failure, as plotted in Fig 7(d). Various cross-sections show that wing flaws initiate at the tips of the pre-existing flaw and propagate a certain distance before stopping. Anti-wing flaws initiate near the tips, propagate a certain distance and stop, coalescing with secondary flaws initiating around the pre-existing flaw, then propagating along the direction of the intermediate principal stress, and eventually coalescing with the specimen’s side surface.

Comparing the results of different test schemes, the failure spatial morphology of specimens with pre-existing through-going flaws is simple, far less complex than that of internal flaws. Confining pressure has a significant influence on flaw propagation, and the influence of the intermediate principal stress on flaw propagation is also clearly visible.

### 5.2. Influence of stress path on the mechanical properties of rock masses with through-going flaws

**Fig 8:**
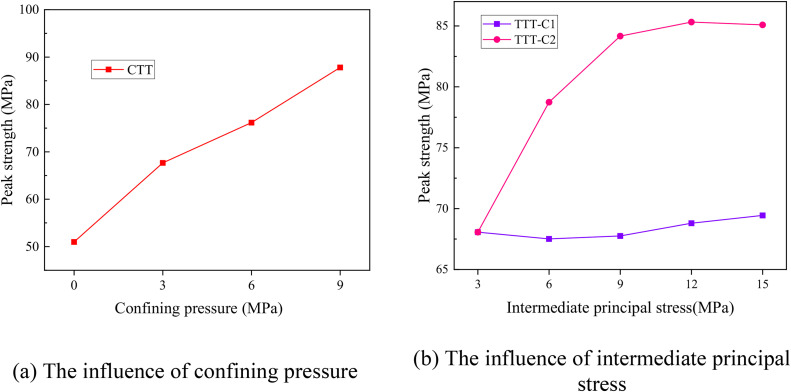
Influence of confining pressure and intermediate principal stress on the peak strength of rock mass with through-going flaws under different stress conditions.

Fig 8 shows the influence of stress conditions on the peak strength of rock specimens with through-going flaws under different stress conditions. Raw data for these plots can be found in S1 File. The results show that the peak strength of the specimen increases significantly with increasing confining pressure, indicating that confining pressure helps to enhance the ultimate strength of the specimen (Fig 8(a)). In the TTT, the peak strength of the TTT-C2 specimens was significantly higher than that of the TTT-C1 specimens, which was consistent with the test results related to their internal cracks. When the intermediate principal stress was perpendicular to the crack plane, increasing the intermediate principal stress within a certain range could improve the peak strength of the specimens. In contrast, when the intermediate principal stress was parallel to the crack plane, increasing the magnitude of the intermediate principal stress showed no obvious enhancement effect on the peak strength. However, increasing the magnitude of the intermediate principal stress after changing its direction could significantly improve the peak strength of the specimens.

## 6. Discussion

### 6.1. Influence of flaw morphology on the failure mechanism of rock masses

**Fig 9:**
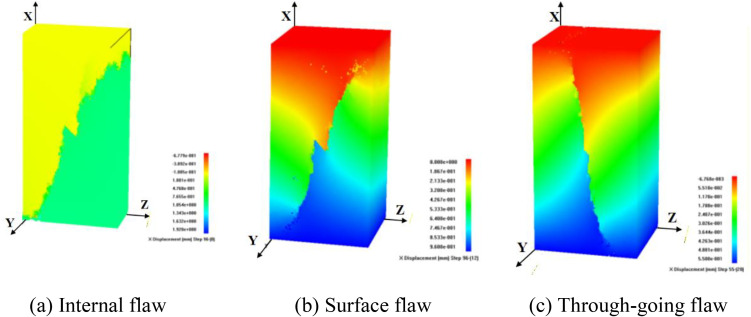
Influence of flaw type on failure mode under uniaxial compression condition.

**Fig 10:**
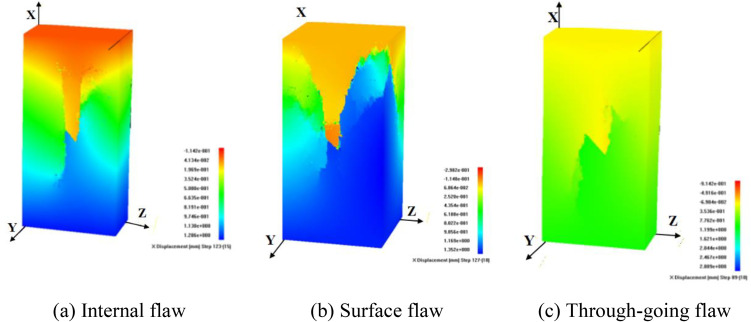
Influence of flaw type on failure mode under conventional triaxial compression condition (σy = 3MPa, σz = 3MPa).

**Fig 11:**
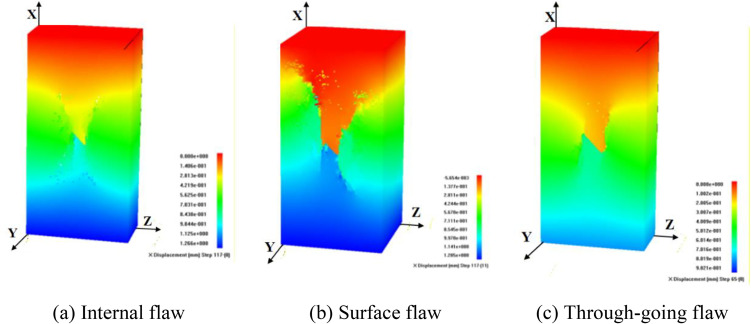
Influence of flaw type on failure mode under true triaxial compression condition (σy = 6MPa, σz = 3MPa).

**Fig 12:**
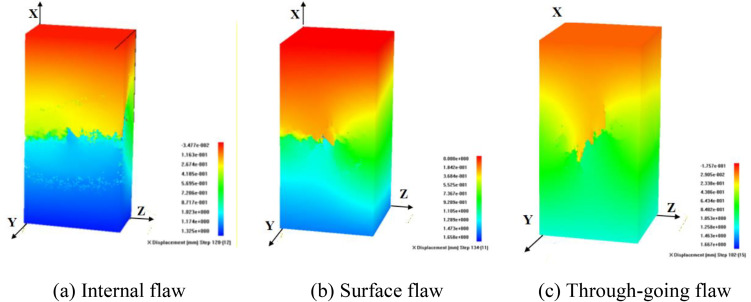
Influence of flaw type on failure mode under true triaxial compression condition (σy = 3MPa, σz = 6MPa).

To analyze the influence of flaw type and stress conditions on the failure mode of specimens, Figs 9-12 present the failure patterns of internal, surface, and through-going flaws under uniaxial, conventional triaxial, and true triaxial loading.

The failure modes of rock masses with different flaws under uniaxial compression are relatively similar. The model ultimately manifests as a shear failure mode that is connected to the structural plane (Figs 9). For specimens with internal and surface cracks, anti-wing flaws are mainly generated at the ends of the cracks and propagate along the direction of maximum principal stress, ultimately leading to shear penetration failure of the specimen (Figs 9(a-b)). For specimens with through cracks, wing flaws are mainly generated at the ends of the cracks and subjected to shear failure along the direction of maximum principal stress (Figs 9(c)).

Under conventional triaxial compression, the morphology of the failure surface is relatively complex due to the influence of confining pressure. For different types of flaws, wing and anti-wing flaws initiate at the tips of the pre-existing crack. With the further increase of load, the cracks propagate and coalesce along the direction perpendicular to the principal stress, leading to tensile-shear composite failure of the specimen (Fig 10).

The failure modes of specimens under true triaxial stress conditions are more complex, and fracture propagation is affected by the direction of the intermediate principal stress. When the intermediate principal stress is oriented toward the flaw plane, the failure modes of the specimens are similar to those observed in conventional triaxial compression tests. For different types of flaws, the specimens generate quasi-“X”-shaped or “Y”-shaped wing and anti-wing flaws, resulting in tensile-shear composite failure of the specimen parameters, as presented in Figs 11.

When the intermediate principal stress is oriented towards the flaw, different types of specimens are dominated by compression-shear composite failure. For specimens with internal and surface flaws, a failure plane perpendicular to the maximum principal stress is generated at the left end, while a tensile failure plane is formed near the right boundary, leading to tension-compression composite failure of the specimens. For specimens with through-going flaws, upward-propagating wing and anti-wing flaws first initiate at the flaw tips, followed by the occurrence of compressive failure, which eventually results in the tension-compression composite failure of the specimens, as illustrated in Figs 12.

The failure behavior of flawed rock masses is governed by the synergistic effects of internal defect characteristics and external stress conditions, and the quantitative differentiation of these influencing factors is crucial for engineering reliability assessment. Therefore, the impact of flaw morphology on rock mass failure mechanisms is subordinate to that of the stress path. Specifically, under uniaxial and triaxial compression, rock masses containing internal, surface, and through-going flaws exhibit typical shear failure as well as tensile or tensile-shear composite failure modes. More importantly, the intermediate principal stress direction relative to the flaw plane is a key controlling factor: parallel alignment induces tensile-shear composite failure, whereas perpendicular alignment leads to compression-shear composite failure.

### 6.2. Influence of flaw morphology on the mechanical properties of rock masses

**Fig 13:**
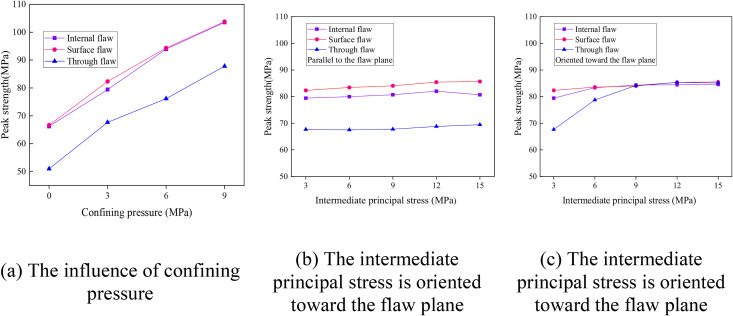
Influence of flaw morphology on the peak strength of rock mass under different stress conditions.

The influence of flaw morphology on the peak strength of rock mass under different stress conditions is shown in Fig 13. The underlying peak strength data for all three flaw types and all stress paths are compiled in S1 File. The results indicate that the peak strength of rock masses increases with the increase of confining pressure. Under the same confining pressure, however, the peak strength of rock masses with surface flaws is slightly higher than that of rock masses with internal flaws, and much higher than that of rock masses with through-going flaws, as illustrated in Fig 13(a).

The effect of the intermediate principal stress orientation on the peak strength of flawed rock masses is relatively complex. When the intermediate principal stress is oriented toward the flaw planes, the peak strength of rock masses with different flaw morphologies remains almost unchanged with the increase of intermediate principal stress, showing a slight upward trend, as shown in Fig 13(b). Under the action of the same intermediate principal stress, the variation law of the peak strength of rock masses with different flaw morphologies is consistent with that under different confining pressures. That is, the peak strength of rock masses with surface flaws is higher than that of rock masses with internal flaws, and much higher than that of rock masses with through-going flaws.

When the intermediate principal stress is oriented relative to the structural planes, the peak strength of rock masses with internal and surface flaws remains almost unchanged with the increase of intermediate principal stress, as illustrated in Fig 13(c). However, the peak strength of rock masses with through-going flaws first increases and then stabilizes with the increase of intermediate principal stress. In addition, under the action of the same intermediate principal stress, the peak strength of rock masses with different flaw morphologies tends to converge with the increase of intermediate principal stress.

### 6.3. Limitations and future work

The current study focuses on quasi-static loading conditions to isolate the coupled effects of stress path and flaw morphology. However, in deep rock engineering, stress paths are often rate-sensitive, and unloading rates significantly influence rockburst potential and large deformations. Future work will include a systematic sensitivity analysis of loading and unloading rates. Additionally, the effect of flaw-to-boundary distance on crack propagation velocity and path will be quantitatively investigated.

## 7. Conclusions

Based on the meso-scale damage numerical simulation method, the failure mechanisms and mechanical properties of three types of three - dimensional fractured rock masses (surface, internal and through-going flaws) under different stress paths were systematically compared and analyzed. The main conclusions are as follows:

(1) The failure mode of flawed rock masses is governed by the coupling effect of flaw morphology and stress path, with stress path playing a more dominant role. Under uniaxial and conventional triaxial compression, the failure modes of all three flaw types are characterized by shear, tensile, or tensile-shear composite failure. Under true triaxial compression, the failure mode is highly dependent on the direction of the intermediate principal stress: when parallel to the flaw plane, tensile-shear composite failure dominates; when oriented toward the flaw plane, compressive-shear composite failure prevails.
(2) The peak strength of all flawed rock masses increases significantly with increasing confining pressure. In contrast, the magnitude of the intermediate principal stress has a relatively weak effect on peak strength. Flaw morphology exerts a more pronounced influence on strength than stress path. Under most loading conditions, surface-flawed specimens exhibit the highest peak strength, followed by internal-flawed specimens, while through-going-flawed specimens show the lowest strength. However, under true triaxial compression with the intermediate principal stress oriented toward the flaw plane at high magnitudes, the peak strengths of the three flaw types tend to converge.
(3) The intermediate principal stress direction critically affects the failure behavior of flawed rock masses. When oriented toward the flaw plane, it promotes transverse crack propagation and enhances peak strength, whereas when parallel to the flaw plane, its influence on strength is less pronounced.

## Supporting information

S1 File Supplementary experimental data and figures. This file contains all raw peak strength data and corresponding plots for rock specimens with internal, surface, and through‑going flaws under different stress conditions, including: (1) influence of confining pressure on peak strength; (2) influence of intermediate principal stress magnitude and direction; and (3) comparative analysis of flaw morphology effects. All data correspond to Figures 4, 6, 8, and 13 in the main text.
(DOCX)
