# Supplementary material for: The influence of stress path and flaw morphology on the failure mechanism and mechanical properties of rock masses
Source: PLoS One. 2026 May 14;21(5):e0349190. doi: 10.1371/journal.pone.0349190 (PMC13175499; doi:10.1371/journal.pone.0349190)
Supplement: S1 File — This file contains all raw peak strength data and corresponding plots for rock specimens with internal, surface, and through‑going flaws under different stress conditions, including: (1) influence of confining pressure on peak strength; (2) influence of intermediate principal stress magnitude and direction; and (3) comparative analysis of flaw morphology effects. All data correspond to Figures 4, 6, 8, and 13 in the main text. (DOCX) [file pone.0349190.s001.docx]

**The relevant data and figures in the paper are given separately.**

1. The Influence of confining pressure on the peak strength of rock mass with internal flaws under different stress conditions

| **Confining pressure/MPa** | **Peak strength/MPa** |
| --- | --- |
| 0 | 66.219 |
| 3 | 79.4245 |
| 6 | 93.920 |
| 9 | 103.544 |

1. Influence of intermediate principal stress on the peak strength of rock mass with internal flaws under different stress conditions

| **Y-load/MPa** | **Z-load/MPa** | **Peak strength/MPa** |
| --- | --- | --- |
| 3 | 3 | 79.4245 |
| 6 | 3 | 79.971 |
| 9 | 3 | 80.693 |
| 12 | 3 | 82.023 |
| 15 | 3 | 80.683 |
| 3 | 6 | 83.338 |
| 3 | 9 | 84.31 |
| 3 | 12 | 84.454 |
| 3 | 15 | 84.549 |

1. The Influence of confining pressure on the peak strength of rock mass with surface flaws under different stress conditions

| **Confining pressure/MPa** | **Peak strength/MPa** |
| --- | --- |
| 0 | 66.6556 |
| 3 | 82.3179 |
| 6 | 94.3377 |
| 9 | 103.808 |

1. Influence of intermediate principal stress on the peak strength of rock mass with surface flaws under different stress conditions

| **Y-load/MPa** | **Z-load/MPa** | **Peak strength/MPa** |
| --- | --- | --- |
| 3 | 3 | 82.3179 |
| 6 | 3 | 83.431 |
| 9 | 3 | 84.041 |
| 12 | 3 | 85.438 |
| 15 | 3 | 85.649 |
| 3 | 6 | 83.56 |
| 3 | 9 | 83.967 |
| 3 | 12 | 85.353 |
| 3 | 15 | 85.501 |

5.The Influence of confining pressure on the peak strength of rock mass with through-going flaws under different stress conditions

| **Confining pressure/MPa** | **Peak strength/MPa** |
| --- | --- |
| 0 | 50.979 |
| 3 | 67.6573 |
| 6 | 76.1538 |
| 9 | 87.7972 |

6.Influence of intermediate principal stress on the peak strength of rock mass with through-going flaws under different stress conditions

| **Y-load/MPa** | **Z-load/MPa** | **Peak strength/MPa** |
| --- | --- | --- |
| 3 | 3 | 67.6573 |
| 6 | 3 | 67.51 |
| 9 | 3 | 67.754 |
| 12 | 3 | 68.798 |
| 15 | 3 | 69.439 |
| 3 | 6 | 78.742 |
| 3 | 9 | 84.167 |
| 3 | 12 | 85.318 |
| 3 | 15 | 85.091 |

7.Influence of flaw morphology on the peak strength of rock mass under different confining pressure

| Flaw morphology  Confining pressure/MPa | **Internal flaw** | **Internal flaw** | **Internal flaw** |
| --- | --- | --- | --- |
|  | **Peak strength/MPa** | **Peak strength/MPa** | **Peak strength/MPa** |
| 0 | 66.219 | 66.6556 | 50.979 |
| 3 | 79.4245 | 82.3179 | 67.6573 |
| 6 | 93.92 | 94.3377 | 76.1538 |
| 9 | 103.544 | 103.808 | 87.7972 |

7.Influence of flaw morphology on the peak strength of rock mass under different intermediate principal stress, which is oriented toward the flaw plane

| **Y-load/MPa** | **Z-load/MPa** | **Internal flaw** | **Surface flaw** | **Through-going flaw** |
| --- | --- | --- | --- | --- |
|  |  | **Peak strength/MPa** | | |
| 3 | 3 | 79.687 | 82.3179 | 67.6573 |
| 6 | 3 | 79.971 | 83.431 | 67.51 |
| 9 | 3 | 80.693 | 84.041 | 67.754 |
| 12 | 3 | 82.023 | 85.438 | 68.798 |
| 15 | 3 | 80.683 | 85.649 | 69.439 |
| 3 | 6 | 83.338 | 83.56 | 78.742 |
| 3 | 9 | 84.31 | 83.967 | 84.167 |
| 3 | 12 | 84.454 | 85.353 | 85.318 |
| 3 | 15 | 84.549 | 85.501 | 85.091 |
